# Time for a New Norm: Experiences of ‘Being Informed’ and ‘Having Choice’ for Prenatal Screening for Chromosomal Conditions: A Qualitative Study

**DOI:** 10.1111/hex.70292

**Published:** 2025-05-19

**Authors:** Niamh Ireland‐Blake, Fiona Cram, Kevin Dew, Sondra Bacharach, Peter Stone, Jeanne Snelling, Christina Buchanan, Sara Filoche

**Affiliations:** ^1^ Department of Obstetrics, Gynaecology and Women's Health University of Otago Wellington New Zealand; ^2^ Katoa Ltd Auckland New Zealand; ^3^ School of Social and Cultural Studies Victoria University Wellington New Zealand; ^4^ School of History, Philosophy, Political Science and International Relations Victoria University Wellington New Zealand; ^5^ Faculty of Medical and Health Sciences The University of Auckland Auckland New Zealand; ^6^ Faculty of Law University of Otago Dunedin New Zealand; ^7^ Auckland City Hospital Auckland New Zealand

**Keywords:** chromosomal conditions, epistemic injustice, epistemic justice, informed choice, prenatal screening

## Abstract

**Background:**

Participating in prenatal screening for chromosomal conditions is premised on an informed choice to accept or decline.

**Aim:**

The aims of this paper are to describe people's experiences of informed choice and how these relate to the experience of prenatal screening.

**Method:**

Thirty‐eight people were recruited and their experiences were explored through narrative enquiry, following an iterative and in‐depth reflexive analysis.

**Findings:**

Informed choice meant ‘being informed’ in ways that met people's cultural needs, values and preferences (e.g., how much information and how it was communicated) and ‘having choice’ (e.g., choice about ‘being informed’, who was involved and choice to enact the decision). ‘Being informed’ affected ‘having choice’. Four themes describe experiences of how informed choice as an ethical principle was upheld: ‘*All I knew it was something that should be done’*, ‘*Going in blind’*, ‘*It would have been frowned upon’* and ‘*I knew I could decline’*. For example, the experience of ‘*I knew I could decline’* describes how the ethical principle of ‘informed choice’ was fully realised. There was a choice about how information was shared that meant people gained knowledge about prenatal screening and a choice about who was involved in this process. A relational experience for ‘being informed’ (e.g., with their pregnancy carer and the decision‐makers) was upheld. People knew that they would be fully supported in enacting their decision. These experiences were not common.

**Conclusions:**

In the absence of ‘being informed’, the possibility for ‘having choice’ is eroded. ‘Having choice’ requires people to have information so that it meets their needs, values and preferences to make sense of it as it relates to their values for decision‐making. Considering ‘being informed’ as an epistemic justice obligation would mitigate eroding the possibility of ‘having choice’. For example, when people experienced prenatal screening as ‘*I knew I could decline’*, it was an epistemically just experience as all the elements for ‘being informed’ for them were met and the relational experience upheld ‘having choice’. The challenge remains for this to be the experience for everyone considering prenatal screening, not just in Aotearoa but globally.

**Patient or Public Contribution:**

The interview questions were reviewed through group discussion with eight people who had lived experiences of prenatal screening for chromosomal conditions. The research was also informed by a researcher who had no lived experience of the screening service as a counter‐view. The premise of this study is to highlight how women and pregnant people experience the consenting process for prenatal screening. The findings may inform organisations, researchers and practitioners about developing approaches for better enabling informed choice in future practice.

## 1 Introduction

Healthcare policy advocates that participation in healthcare should reflect ‘informed choice’ [1, 2, 3]. Informed choice enables a person to exercise autonomy, which is regarded as central to ethical practice. A broad definition of informed choice is one where people have been given the relevant information, and the decision to have, or not have, screening is consistent with their beliefs and values [1]. An explicit policy aims to promote informed choice to enhance patient autonomy (the right to make choices) and to prevent people from being deceived or coerced [1].

Participating in prenatal screening for chromosomal conditions has been part of antenatal care in many countries for over 40 years [3]. In many countries, including in Aotearoa New Zealand (Aotearoa), where this study was carried out, prenatal screening for chromosomal conditions is optional [2]. Pregnancy care in Aotearoa is provided by a lead maternity carer (LMC), who is most often a midwife. Depending on the trimester of the pregnancy when people register with an LMC, prenatal screening for chromosomal conditions occurs either in the first or second trimester. Information about prenatal screening is usually provided at people's first antenatal visit with their LMC, along with other information about aspects of their pregnancy care.

Promoting reproductive autonomy is recognised as one of the core principles of prenatal screening for chromosomal conditions [3]. Enabling informed choice minimises the potential for routinisation (i.e., where an option becomes the expected norm) [2, 3, 4]. In Aotearoa, and elsewhere, professional guidelines state that pregnant people need to be informed about the option for prenatal screening for chromosomal conditions and that cultural and linguistic diversity are considered [2]. How people are supported to make informed choices about their healthcare is therefore an epistemic consideration. Much research to date has focused on measuring informed choice, knowledge of prenatal screening, experiences of the screening procedure, interventions to increase knowledge about prenatal screening or decision aids to facilitate decision‐making processes [5, 6, 7, 8, 9, 10]. There is a gap in the literature about people's experiences of ‘being informed’ and ‘having choice’. Mapping these experiences could provide new insights into achieving informed choice in future practice. The aims of this paper are to describe people's experiences of informed choice and how this relates to informed choice being upheld in the experience of prenatal screening for chromosomal conditions. These experiences were gathered through narrative enquiry, which supports deep understanding of human experiences, particularly those that are complex and nuanced [11, 12].

## 2 Materials and Methods

### 2.1 Ethics Approval

Ethical approval for this study was granted by the Health and Disability Ethics Committees (21/STH/82).

### 2.2 Participants and Recruitment

For this qualitative research project, a total of 38 people were recruited for pragmatic reasons (e.g., length of project and funding). We prioritised the inclusion of Indigenous (Māori) participants. Thirty‐seven people were recruited through a market research company (Qantar) from three different locations in Aotearoa (Christchurch, Auckland and Wellington). One person was recruited through a snowball approach. People were eligible to participate if they were 18 years of age and older, were not currently pregnant and had been pregnant in the last 3 years. The recruitment company contacted potential participants and shared the study information with them. Time for discussion was arranged, and SKF emailed the participants an introduction to confirm the appointment time. The recruitment company had no access to the information shared during the discussion. As an acknowledgement, participants were given a $50 (NZD) gift voucher.

### 2.3 Researcher Position and Discussion Prompt Questions

The authors are Indigenous (Māori) and non‐Indigenous researchers with experience in maternal foetal medicine, genetic counselling, bioethics and law, epistemology and medical sociology. Prompt questions aimed to explore experiences and qualities essential to being informed and deciding to have prenatal screening (Table 1). The questions were developed by the authors (except for NI‐B). The questions were reviewed through group discussion with eight people who had lived experiences of prenatal screening for chromosomal conditions. The research was also informed by a researcher who had no lived experience of the screening service as a counter‐view. The prompt questions were piloted with five people who had recently been pregnant.

**Table 1:** Examples of the questions used to guide the discussion.

If you think about the offer of prenatal screening, what worked (or didn't) for you? If you think about informed choice—what does that mean to and for you? How did you make a decision to accept or decline prenatal screening? ○ Did you involve anyone else in discussing this/deciding? ○ Did you feel that you had enough time? ○ Do you feel that you had an opportunity to make an informed choice? What would help you think/feel/know that you have made an informed choice? What did you talk about? ○ Did you feel comfortable asking questions/seeking clarification? ○ Did you feel like you were listened to and your concerns taken seriously? What would a more affirming consent process look like for you? Is there something else that you would like to add?

### 2.4 A Guided Discussion Through Narrative Enquiry

A guided discussion using narrative enquiry was used. As with other forms of qualitative research, narrative enquiry allows thematic patterns to emerge when exploring the lived experiences of individuals. This approach allows for dialogue and cyclical reflection. Time was taken to make introductions, say a prayer/karakia and explain the project. All the discussions were held in English and facilitated by SKF (European descent, healthcare scientist and mother of two). As the discussions took place, SKF aligned the scope of discussion with what wāhine and women knew about screening and how they wanted to discuss their experiences. SKF also shared her experiences of prenatal screening, and often the discussions became akin to an exchange of stories and experiences. These were genuine exchanges. Due consideration was practiced so that the voices and experiences of the participants were prioritised. All the discussions were audio recorded with participant permission.

The discussions lasted between 30 min and 1 h and finished with prayer/karakia as appropriate. Participants were asked if they would like a copy of their audio transcript and/or a copy of the final findings. All of the discussions were held online (Zoom) or by phone due to Covid‐19 restrictions at the time.

### 2.5 Approach and Reflexive Thematic Analysis

Grounded theory framed the approach to enable an inductive process whereby theoretical insights were generated from the transcripts [13]. Grounded theory allows emergent themes and concepts to drive the process, rather than starting with pre‐defined hypotheses [13]. People's accounts were professionally transcribed and cross‐checked with the audio recordings by SKF and NI‐B. The qualitative coding software NVivo was used to support the analysis (Lumivero, Release 1.0, 2020). A reflexive thematic analysis was employed to analyse the narrative discussion data. We analysed the narratives, viewing the shared stories as data, which we then analysed for recurring themes [11, 12]. The themes were derived iteratively based on the verbatim transcripts, our overarching aim being to describe the emerging range of experiences without pre‐conceived assumptions [14, 15]. Adopting a reflexive approach enabled us to consider our position in the findings and to consider how the resultant themes would be framed [11, 12]. Through the reflexive approach, themes are not predefined to ‘find’ codes. In reflexive analysis, themes are produced by organising codes around a relative core commonality, or ‘central organising concept’, that we, as the researchers, interpreted from the data [14]. Initial codes were derived, and subsequent themes were initially independently developed by SKF and NI‐B, and then through an iterative process involving five rounds of analysis and discussion, the final themes were derived and named by consensus (Table 2) [14, 15].

**Table 2:** Coding frame and thematic structure.

Inductive codes	Secondary codes	Preliminary themes	Final themes
Negotiated	All the time in the world	Informed and choice	‘*I knew I could decline’*
	Not just a Māori way for Māori	Connectedness and informed choice	
	She was whānau		
	How she made us feel		
Not negotiated	A Māori midwife would have made a difference	Relationships with unmet connections	‘*All I knew was that it was just something that should be done’*
	Going in blind		‘*Going in blind’*
	I was lucky		‘*It would have been frowned upon’*
	Scary		
	Power	What would have worked	‘*I get there's a lot of information, but I'd rather be overwhelmed than underwhelmed’*
	Expected to do it		‘*If one person is informed a whole whānau's informed’*
	Can't get information		
	Not a conversation		

## 3 Results

Thirty‐eight people shared their experiences of being informed about prenatal screening from three geographic areas in Aotearoa (Christchurch, Auckland and Wellington). Eighteen participants identified as Māori; 8 as New Zealand European; 6 as Indian; 2 as Chinese; 1 as Chinese/European; 1 as Asian; 1 as Canadian and 1 as Pacific Peoples. Thirty‐six wāhine (Māori women) and women had prenatal screening.

Having information did not equate to having a choice. Informed choice meant ‘being informed’ in ways that met people's needs, values and preferences (e.g., what and how much information and how it was communicated) and ‘having choice’ (e.g., choice about these information preferences, who was involved and choice to enact the decision). Everyone's needs for fulfilling these elements, and the levels to which they were met, varied. Often, the choice of LMC was down to ‘luck’ as to who was available, especially for whānau Māori (Māori family) wanting a Māori LMC.

### 3.1 Presentation of the Findings

The findings are presented in two main sections (Table 2): ‘Experiences of informed choice’ and ‘Thoughts for future practice’. The first section describes how experiences of ‘being informed’ affected ‘having choice’, and consequently, how this affected informed choice as an ethical principle was, or was, not upheld. The second section describes people's experiences of ‘being informed’ and reflections about how informed choice could be better met in future practice.

### 3.2 Experiences of Informed Choice

#### 3.2.1 ‘All I Knew Was That It Was Just Something That Should Be Done’

The theme ‘*All I knew was that it was just something that should be done’* describes how ‘being informed’ and ‘having choice’ were not features of people's experiences. When ‘being informed’ was missing from people's experiences, screening was described as the expected norm, that it was ‘*part of the process’* and, something that people ‘just did’, and often, felt like they had to. In these instances, the right to make an informed choice was not upheld.

‘…all I knew was that it was just something that should be done.’

In reflecting on their experience, one person shared,

‘Clearly we've consented to something we haven't known about.’

As a result, it often meant that people felt unprepared; they didn't know what to expect from the ultrasound or what was involved in the blood test, and sometimes what conditions were being screened for.

‘I would've liked to know, yeah, what it was, and I would've liked to be told more about it because you don't know what you're going there for. Then I would've prepared myself more … yeah, unprepared sucks.’

People often felt lucky in these experiences that nothing had been detected and that it would have been a shock if something had been detected due to being unprepared going into the screening.

#### 3.2.2 ‘Going in Blind’

The theme ‘*Going in blind’* describes an experience of when ‘being informed’ was founded on information that hadn't met people's needs, beliefs, values or preferences as it related to their decision‐making. As a result, this eroded the element of ‘having choice’ and consequently, informed choice was not realised. This theme represented most people's experiences.

‘You just get overwhelmed with information but I guess, well 'cause you're so blinded by it, I kind of was just going in blind following my midwife.’

In these experiences, when the element of ‘having choice’ was eroded, people often described how much trust they felt they needed to place on, or was assumed by, their LMC because they were ‘*the professional’*.

‘That's not informed consent. That's just “I trust you to be doing the right thing for me” consent.’

When people experienced ‘*it was just something that should be done*’ or ‘*going in blind*’, it was often synonymous with a transactional experience with their LMC. Wāhine and women felt they were ‘*just a number*’ going through a ‘*shopping list*’ or a ‘*tick‐box*’ exercise.

‘My midwife was all business and didn't really tell me anything. Just said, “this is how it is, I'm going to book this doctor's appointment, give me your name, and all your details,” and then shut the laptop, and she was gone.’

Experiences of ‘*all business*’ like interactions had the effect of limiting opportunity for ‘being informed’ and for further discussion. People described ‘*just going quiet’*, as they hadn't felt heard or would not have been listened to if they had asked questions. For others, if they weren't the kind of person to ask questions, there wasn't space created for them to be, or feel, encouraged to do so. Some people felt like they had to demonstrate credibility because they felt that they were seen as ‘*a silly first‐time mum*’ who had ‘*watched too many TikToks*’. Others felt that assumptions were made about what they did (but actually didn't) know because they were older or that it wasn't their first pregnancy. Some people felt that they had to present themselves in a certain way to be taken more seriously, such as wearing their work clothes and lanyards to appointments. If wāhine and women were given space to ask questions, they often didn't know what questions to ask as they “didn't know what they didn't know”.

“We'd just say ‘we don't know what questions to ask 'cause we don't know what we're asking about.’”

For some wāhine and women, a ‘business’ model of care was something that worked for them. Others wished (and had tried) for a more relational experience with their LMC, as they would have felt more at ease to have asked questions without feeling like they were the ‘wrong’ ones to raise.

These initial experiences often resulted in a trajectory of limited or no opportunity for ‘being informed’ in further aspects of pregnancy care.

‘You're kind of set up to not ask questions. You know, you kind of just “okay you're going to do this” and then so you just go “okay I guess I am.” Then the next time they say “you're gonna do this,” and you're like “okay I just do what you say.” It's not a conversation opener, or you don't feel like “oh what is that, can I ask about that?” It's a “okay you're the professional here I'm not, so your word is gospel 'cause I don't know any better.”’

Wāhine and women recognised that a ‘business’ approach was, at times, attributed to the demanding workload of their LMC.

#### 3.2.3 ‘It Would Have Been Frowned Upon’

‘*It would have been frowned upon’* describes how, even when the element of ‘being informed’ was met, the element of ‘having choice’ was eroded due to wāhine and women being unable to enact their decision to undergo screening based on the decisional factors important to them. For example, some wāhine and women understood what screening entailed and knew that it was optional, but felt that they didn't have the option to decline.

‘I think that I would have been able to decline but I would have felt as though it would have been frowned upon.’

When informed choice was experienced as ‘*it would have been frowned upon’* people felt unsure about whether they and their decisions would be supported by their LMC and therefore did what was expected of them (or what they felt was expected of them). As a result, informed choice was not fully realised. To feel supported in their choices, people needed their experience of having the choice to be more relational.

#### 3.2.4 ‘I Knew I Could Decline’

‘*I knew I could decline’* describes how the ethical principle of ‘informed choice’ was fully realised. People were informed in ways that met their cultural needs, values and preferences and had the choice (autonomy) to accept or decline screening.

‘I knew I could decline.’

When wāhine and women experienced informed choice early in their pregnancy, they felt empowered and had autonomy in the decisions that they and their whānau made about other aspects of their pregnancy and care.

‘They never once said, you know, you have to have this. They always said, you know, if you are informed and you understand your results, you're always gonna make better choices.’

‘Being informed’ and ‘having choice’ experienced as ‘informed choice’ was described in the context of experiences founded on deeply felt trust of the relationship with their LMC.

‘She's [LMC] the one that we're gonna go with purely because of how she made us feel. So when it came to informed choice she basically read through anything and everything and broke down the jargon … she went through every single thing with us and just went over and over and happily answered those questions … that was a big thing for my husband and I was us feeling comfortable with the midwife. We had so much trust in her to help guide us. She said “right I'm not getting you to just go and get bloods for the sake of getting bloods, this is what it's for, this is why, if you don't want to do it you don't…” like she always said “if you don't want to do something you don't have to do it.” But she would explain why, and then she went through it…. It was definitely that first meeting with her as our midwife which kind of set‐in stone that yeah, this is someone that we could trust.’

Through a process of ‘being informed’ in a way that met people's needs and values, the relational experience was deepened. The ‘right’ people were involved in the process, and they, as the decision‐makers, knew that they would be fully supported in enacting their decision to have screening or not.

#### 3.2.5 Thoughts for Future Practice

The theme ‘thoughts for future practice’ describes people's reflections on ‘being informed’ and how this could have been met differently for them and for others in the future.

#### 3.2.6 ‘I Get There's a Lot of Information, but I'd Rather Be Overwhelmed Than Underwhelmed*’*

The theme, ‘*I get there's a lot of information, but I'd rather be overwhelmed than underwhelmed’*, describes how people's values and preferences for being informed were variably met. Qualities that were emphasised for ‘being informed’, meeting people's values and preferences, included ‘*calming’, ‘thorough’*, ‘*responsive’* and ‘*guided’*.

Often, wāhine and women were given a pamphlet, which came with a ‘*pile*’ of other information that just got ‘*put in a drawer*’. Sometimes wāhine and women had to go out of their way (beyond their pregnancy carer) to acquire the information they needed. This included online searches with ‘*Aunty Google*,’ or that information was shared through work colleagues, whānau/family or friends. Some people wanted and expected the information to come only from their pregnancy carer, as they were seen as their ‘*source of everything*’.

Of the two people who declined screening, one did so because by the time they had the information they needed, which they had to navigate themselves, it was too late to have the screening, with no opportunity for choice. But, because they now understood what screening entailed, it was something that they would consider in the future.

‘I ended up having to Google it afterwards and I was just like, they [pregnancy carer] should really tell you this. But it was just slightly terrifying as well. They [LMC] were like, “Down syndrome could run in your family you should really get a checked.” I'm just like “Well I would if you told me what actually goes on, like do I get stabbed, do I get operated on, what happens?” But it took two turns of Google to actually find out, and at that point like the opportunities would have passed. But I'd probably look at it for future ones [pregnancies]’.

Peoples' reflections of how ‘being informed’ could have been (better) for them would have meant having the choice about how information was provided, what and how much, and who needed to have been involved (i.e., relational). People were rarely asked about these preferences. So while some people were ‘blinded’ by all the information, when they would have preferred the ‘highlights’, for others, this would have met their preferences.

‘I get there's a lot of information, but I'd rather be overwhelmed than underwhelmed.’

‘Being informed’ and ‘having choice’ as relational experiences didn't always rely solely on the LMC. Sometimes it meant involving other people (e.g., partners) outside of the appointment where prenatal screening was discussed.

‘I would actually, in some cases, force him [husband] to read the information, going, “No, you need to know this because this is for our child. It's not just my child.”’

For many others, it meant involving others (e.g., partners/whānau) in the appointment. Sometimes, due to Covid‐19 restrictions at the time of their pregnancy, involving others was not always possible, or when they were at the appointment, not facilitated.

‘I still really remember that the conversation was between me and my midwife and I didn't feel like my husband had an opportunity to, he wasn't consulted, it was like the decision was mine only. It's not me that's carrying this decision, it's me and him and you know, the whānau [family] as well. And I think that needs to be really explored because the outcome of those tests are a whānau's to carry.’

As the above experiences indicate, relational experiences are integral to people's values and preferences for informed choice because the outcome of screening will be managed relationally.

#### 3.2.7 ‘If One Person Is Informed, a Whole Whānau Is Informed’

The theme ‘*If one person is informed, a whole whānau is informed’* describes people's reflections for informed choice for whānau Māori (Māori family(ies)) in the future. Receiving care that upheld cultural needs was an important element of informed choice, but it was not always met.

Two whānau Māori had a Māori LMC. One wāhine and her whānau took on a non‐Indigenous LMC with more midwifery experience ahead of a Māori LMC with less, as they had had previous pregnancy complications and were confident in holding space for tikanga (Māori customary practices, values and protocols) themselves. Others, however, felt that they would have preferred having an LMC who could better guide and uphold tikanga for them, but struggled to find one. Many wāhine Māori thought that they would have had more opportunity for informed choice to be supported through deeper relational connections and trust with a Māori, or Pacific LMC. For others, being culturally informed didn't always mean that wāhine needed a Māori LMC, rather someone who understood the importance of elements such as trust and connection if ethnicity was not in common.

‘They [LMC] don't even necessarily need to be Māori, but to understand that you're dealing with Māori or Islander people that might want to know that that option's available.’

or

‘You have trust in this LMC regardless of their ethnicity … my experience with dealing with Māori … you have more of that, more things in common, so the conversations are easier and there's more trust there than with someone who is of a different ethnicity … you already have that level of built‐in trust.’

Having conversations and discussions with other whānau Māori at marae (meeting house), or other safe and familiar, community‐based places, would uphold relational ways of sharing information and cultural norms for acquiring and developing knowledge about prenatal screening.

‘I think more Māori community based, marae [meeting house] based programmes for hapū [pregnant] māmā and stuff like that to actually allow the sharing of information, the sharing of kōrero … the ability to sit down and actually just have a kai, have a cup of tea … delivered by our own to our own, or by people that understand the group that they are presenting to you. You have to have ngākau Māori, you have to have that Māori heart, that Māori understanding to be able to communicate in a way that's (A) cultural appropriate, (B) understandable and comfortable.’

Making information about prenatal screening more widely available, beyond clinical spaces, would also increase opportunities for relational ways of sharing information and cultural norms for acquiring and developing knowledge.

‘I think it [screening information] should be made available at the kohangas [Māori medium pre‐school] and also at like the marae and like community centres because like if it's sitting there at like, at the marae front desk, someone's gonna pick it up and read it, and if one person is informed, that means a whole whānau's informed.’

## 4 Discussion

Midwifery models of care, both in Aotearoa and elsewhere, support relational and holistic care that sees pregnancy as normal, non‐medical [4]. At the time of this project, the midwifery workforce in Aotearoa was under considerable pressure and continues to be. Increasing the number of Indigenous maternity carers (and for mainstream models of maternity care to be more culturally informed) was highlighted and is a recognised workforce need [16]. Our research is not intended to place blame; rather, it upholds people's experiences as shared to support informed choice for prenatal screening for other people in the future.

Participation in population screening programmes is voluntary based on people's informed choice to accept or decline screening. This paper aimed to explore people's experiences of informed choice in the context of prenatal screening through a narrative enquiry approach and in‐depth reflective thematic analysis. Our findings showed how the experience of ‘being informed’ affected ‘having choice’ and consequently, how informed choice as an ethical principle was upheld. Having information did not equate to having a choice [17]. Our findings showed that informed choice meant ‘being informed’ in ways that met people's needs, values and preferences. That is, what information, in terms of the level of detail, how much information in terms of amount, and where and how it was communicated. It also meant ‘having choice’. That is, the choice about how these informational preferences were met, who was involved, the choice of pregnancy carer and the choice to enact the decision. Everyone's needs for fulfilling these aspects of ‘being informed’ and ‘having choice’, and the levels to which they were met, varied. When people experienced ‘*all I knew that it was something that should be done’* or ‘*going in blind’,* the possibility of ‘having choice’ was eroded because the element of ‘being informed’ was not (fully) met. Our findings showed the importance of *how* information was conveyed and how this did, or didn't, meet people's needs for making sense of it as it relates to their values for decision‐making. These findings extend our present understanding of informed choice as more than an epistemic consideration to one of epistemic justice.

Epistemic justice is when people's positions as a knower and producer of knowledge are upheld [18, 19]. Epistemic justice considers people's epistemological norms (e.g., people's different and varied worldviews and ways of acquiring knowledge), social justice (e.g., human rights and equity) and ethical practice (e.g., autonomy) [20]. There are two ways that this can broadly occur. Hermeneutical justice is upheld when people have, for example, the resources and language to express and make sense of the experience to, and for, them [18, 19]. Testimonial justice occurs when people's testimonies of their experience are believed as being credible [18, 19]. The converse occurs in instances of hermeneutical and testimonial injustices. Epistemic injustices are inextricable from injustices associated with race, gender and socioeconomic status [19, 20]. Informed choice as an epistemic justice consideration has not been widely explored, but it promises new avenues for clinical communication approaches and policy implications [20, 21, 22].

In the context of people's experiences of informed choice, and the resultant experience of informed choice as an ethical principle, epistemic (in)justices can be seen in several different ways. When people experienced prenatal screening as ‘*I knew I could decline’*, it was an epistemically just experience. From a hermeneutic perspective, it meant that information was shared and available in ways that met values, preferences and needs, so people acquired knowledge of, and about, prenatal screening. There was also a choice about ‘being informed’, for example, who was involved in the discussion and decision. From a testimonial perspective, the discussion upheld people's credibility for knowing. People were also supported to enact their decision. The ethical principle of informed choice was upheld [2].

These were not everyone's experiences, nor for many others that are echoed in other countries spanning more than 20 years [23, 24]. Most recently, in a US‐based project, people were offered prenatal screening for a range of genetic conditions [25]. They shared experiences such as ‘*I was left in a position where I was like, I don't even know what any of this means. Then I turned to Dr Google, which I do not recommend*’ [25]. Another person from the same project shared, *‘Maybe if someone would've went through with me, and really explained it, maybe that would've made it a little bit easier to process’* [25]. These examples also highlight the importance of considering the hermeneutics of informed choice. Standardised information about prenatal screening, for example, pamphlets, is the usual practice. It can support information needs, particularly in time‐constrained settings [26, 27]. However, a standardised approach has the potential to perpetuate hermeneutical injustices as there is an embedded expectation to conform to normative, often biomedical ways of knowing [26, 28]. This is not to say that biomedical knowledge is not important, but not to the exclusion of other ways of knowing.

Some people we spoke with shared that they felt that negative assumptions were made about them and their credibility as knowers, or for knowing. Some felt expectations of knowledge because it wasn't their first pregnancy, or that they were being stereotyped because of their physical appearance, or that they had to dress smartly to be taken seriously. If people are misattributed as not being credible for knowing due to stereotypes, these are types of testimonial injustices [19, 20]. In these instances, the exchange of information may be impeded, affecting how, what and if information is shared [18, 19, 20]. Testimonial injustices have been explored in other areas of prenatal screening as they relate to how people's experiences of living with a chromosomal condition (e.g., Down syndrome) or parents of someone living with a chromosomal condition are not often privileged in information provided to prospective parents [26, 29]. An epistemically just process would help mitigate any such experiences as it would consider ways of knowing beyond the current norm and be free from judgement that sees everyone as credible for knowing.

Our findings have highlighted the importance of the relational experience as it purports to inform choice. Information sharing involves exchanging knowledge, expertise and insights among individuals, or groups of people, to support decision‐making processes [30, 31, 32]. This is an epistemic justice consideration as it acknowledges people's epistemological norms and therefore supports opportunities for ‘having choice’. People we spoke with described ‘being informed’ and ‘having choice’ in the context, and the construct, of relationships. Often these were beyond dyadic interactions with their pregnancy carer, to ones more aligned with relational autonomy [4, 29, 33]. That is, that ‘being informed’ and the decision (and any outcome of screening) was considered in terms of relationships with a partner, family and whānau. The relational experience that people had with their pregnancy carer shaped how they were supported in ‘having choice’ (e.g., ‘*It would have been frowned upon’*). It also supported the relational exchange of information, how people felt, whether or not they were informed and how this supported the involvement of others, such as whānau. These early experiences also shaped how people felt that they were able (or not) to ask questions, and have a choice, over other aspects of their pregnancy. As experienced by wāhine and women, demanding workloads affected how relationships were established and availability to facilitate communicative spaces. Structural considerations are important to this (e.g., time available for appointment, workloads, etc.), and these are unlikely to change in the immediate future. However, there are possibilities for changing the current norm. In terms of supporting the relational experiences for sharing information, it doesn't necessarily need to happen at the time of the consultation or in a clinical setting. To the extent that these spaces may be better created at other times and in safe and familiar places and spaces. For example, wāhine shared that having conversations and discussions with other whānau Māori at marae, or community‐based places, would uphold cultural norms. If we continue to consider informed choice devoid of the relational experience, the risk of eroding ‘having choice’ will persist and the opportunity to redress existing epistemic injustices will be limited.

### 4.1 Strengths and Limitations

The main strengths of this study are the rich experiences of informed choice for prenatal screening for chromosomal conditions that were shared. Representation was ethnically diverse, from a range of social backgrounds and from three geographic regions in Aotearoa. Potential limitations include that the conversations were had in English, with one person, and were held online/phone. Inclusion of other geographical areas and recruitment through other avenues may have also yielded more positive experiences of informed choice. Notwithstanding these limitations, our findings show intersectional generalisability, in that they mirror and build on other experiential research that has explored informed choice in other countries. The variation in experiences reflected the variation in how people's experiences of informed choice were upheld [34, 35]. We did not explicitly ask about information preferences in terms of content or determining what screening information people consider to be relevant to, or for, them. Ascertaining the relevance of the information provided is certainly an important consideration, particularly as this has not been widely explored in the context of prenatal screening in Aotearoa.

## 5 Conclusions

Our findings echo those of others that span more than 20 years, in which people's experiences of prenatal screening have been described as an expected norm [23, 24]. If this is to change, a new norm is needed. Considering ‘being informed’ as an epistemic justice obligation would mitigate eroding the possibility of ‘having choice’ and bring us closer to realising the ethical principle of informed choice.

## Author Contributions

**Niamh Ireland‐Blake:** writing – original draft, writing – review and editing, data curation, formal analysis. **Fiona Cram:** conceptualisation, investigation, funding acquisition, writing – original draft, methodology, writing – review and editing, formal analysis. **Kevin Dew:** conceptualisation, investigation, funding acquisition, writing – original draft, methodology, writing – review and editing. **Sondra Bacharach:** conceptualisation, investigation, funding acquisition, writing – original draft, writing – review and editing. **Peter Stone:** conceptualisation, investigation, funding acquisition, writing – original draft, writing – review and editing. **Jeanne Snelling:** conceptualisation, investigation, funding acquisition, writing – original draft, writing – review and editing. **Christina Buchanan:** conceptualisation, investigation, funding acquisition, writing – original draft, writing – review and editing. **Sara Filoche:** conceptualisation, investigation, funding acquisition, writing – original draft, methodology, writing – review and editing, formal analysis, project administration, supervision, data curation.

## Ethics Statement

Ethical approval for this study was granted by the Health and Disability Ethics Committees (21/STH/82).

## Consent

All participants provided written consent.

## Conflicts of Interest

The authors declare no conflicts of interest.

## Data Availability

The data are not available for use as per the ethics approval obtained for the research.
